# Low- and High-Cycle Fatigue Behavior of FRCM Composites

**DOI:** 10.3390/ma14185412

**Published:** 2021-09-18

**Authors:** Angelo Savio Calabrese, Tommaso D’Antino, Pierluigi Colombi, Carlo Poggi

**Affiliations:** Department of Architecture, Built Environment and Construction Engineering, Politecnico di Milano, 20133 Milan, Italy; tommaso.dantino@polimi.it (T.D.); pierluigi.colombi@polimi.it (P.C.); carlo.poggi@polimi.it (C.P.)

**Keywords:** FRCM, TRM, TRC, fatigue, low-cycle fatigue, high-cycle fatigue, tensile test

## Abstract

This paper describes methods, procedures, and results of cyclic loading tensile tests of a PBO FRCM composite. The main objective of the research is the evaluation of the effect of low- and high-cycle fatigue on the composite tensile properties, namely the tensile strength, ultimate tensile strain, and slope of the stress–strain curve. To this end, low- and high-cycle fatigue tests and post-fatigue tests were performed to study the composite behavior when subjected to cyclic loading and after being subjected to a different number of cycles. The results showed that the mean stress and amplitude of fatigue cycles affect the specimen behavior and mode of failure. In high-cycle fatigue tests, failure occurred due to progressive fiber filaments rupture. In low-cycle fatigue, the stress–strain response and failure mode were similar to those observed in quasi-static tensile tests. The results obtained provide important information on the fatigue behavior of PBO FRCM coupons, showing the need for further studies to better understand the behavior of existing concrete and masonry members strengthened with FRCM composites and subjected to cyclic loading.

## 1. Introduction

The 2030 Agenda and its Sustainable Development Goals are determining a distinctive change in route for the construction sector [1]. Besides the traditional focus on new constructions, strengthening, retrofitting, and revalorization of existing structures is gaining increasing importance. Within this field, a fundamental role is played by the employment of low environmental impact and durable materials, which shall guarantee quality, durability, and sustainability of the applications. Among these materials, fiber-reinforced inorganic-matrix composites, which are comprised of non-metallic high-performance fiber textiles embedded within inorganic matrices, are a valid solution for strengthening and retrofitting existing masonry and concrete members. These composites are usually referred to as fiber-reinforced cementitious matrix (FRCM) [2,3], textile reinforced mortar (TRM) [4,5], or textile reinforced concrete (TRC) [6,7] when the matrix is a high-performance finely grained concrete. In this paper, the term FRCM is used. Externally bonded (EB) FRCM are a lightweight, non-invasive, durable, and cost-efficient strengthening/retrofitting solution, reportedly effective in increasing the bearing and displacement capacity of structural members and reducing the crumbling/overturning hazard of non-structural members [8,9,10,11,12,13].

Studies recently conducted by the authors on FRCM composites applied to the tension side of reinforced concrete (RC) beams showed their effectiveness in increasing the fatigue life of members subjected to cyclic actions, such as in bridges and viaducts, which due to their intended use, are subjected to fatigue cycles induced by the vehicular traffic [14,15]. This increase in the fatigue life is due to the composite capability to reduce the tensile stress acting on steel rebars under service loads of a different extent depending on the beam geometry and composite properties [15]. Besides, the presence of the FRCM composite limits the beam deflection and determines thinner and more spaced flexural cracks in comparison with nominally equal unstrengthened RC beams [16]. This improves the fatigue performance of the beam and its dissipative capacity under seismic actions. Furthermore, the presence of thin cracking reduces the rebar exposure to an aggressive environment with respect to wide cracking, which increases the steel durability and fatigue life [17,18]. The experimental evidence showed increases in the strengthened beam fatigue life up to seven times if compared to that of corresponding unstrengthened beams [15]. However, this effectiveness was linked to the bond between FRCM and substrate and to the composite fatigue performances.

In this paper, the cyclic behavior of a hybrid PBO-Glass FRCM composite was experimentally investigated by tensile testing of composite coupons using the clamping-grip method [19]. The effect of low- and high-cycle fatigue on the composite tensile strength, ultimate strain, and slope of the stress–strain curve was investigated. Besides, the influence of fatigue cycles on the formation of matrix cracks and on their widening and propagation, as well as the energy dissipated in each cycle, was analyzed. For some specimens, post-cyclic tests were performed to study the effect of low-cycle fatigue loading on the specimen residual tensile strength, ultimate strain, and slope of the stress–strain curve.

## 2. Materials and Methods

The hybrid PBO-Glass FRCM composite investigated in this paper was constituted of one layer of textile embedded between two layers of a cement-based matrix, each 5 mm thick [20]. The textile was unbalanced, with PBO bundles along the warp direction (i.e., aligned with the load direction and parallel to the specimen longitudinal axis) and alkali resistant (AR) glass bundles in the weft direction [21]. Warp yarns were spaced at 10 mm on center, whereas weft bundles at 25 mm on center. The cross-sectional area of a single longitudinal PBO yarn was *A_f_* = 0.56 mm^2^ (nominal width and thickness of b* = 5 mm and t* = 0.11 mm, respectively, equivalent thickness of the textile *t_f_* = 0.056 mm). The average tensile strength σ_fu_, ultimate strain ε_fu_, and elastic modulus *E*_f_ of the PBO textile in longitudinal direction were obtained by tensile testing of nine 50 mm wide and 400 mm long bare (i.e., not impregnated) textile strips, according to the indication of the European Assessment Document (EAD) for FRCM composites [22]. The bare textile strips included five longitudinal yarns and were equipped with an extensometer with 50 mm gauge length to measure the strain during the test. The results obtained are reported in Table 1, along with the average compressive and flexural strengths of the cement-based matrix, experimentally obtained by testing 12 samples according to UNI EN 1015-11 [23]. The matrix tensile strength σ_mu_ and elastic modulus *E*_m_ in Table 1 were taken from [24].

Twenty-eight FRCM coupons were realized in this study, following the indications of the EAD for FRCM composites [22]. The coupons had nominal dimensions equal to 400 mm (length), 50 mm (width), and 10 mm (thickness). Each specimen included 5 longitudinal PBO yarns, which resulted in an overall longitudinal fiber cross-sectional area *A* = 2.80 mm^2^.

**Figure 1:**
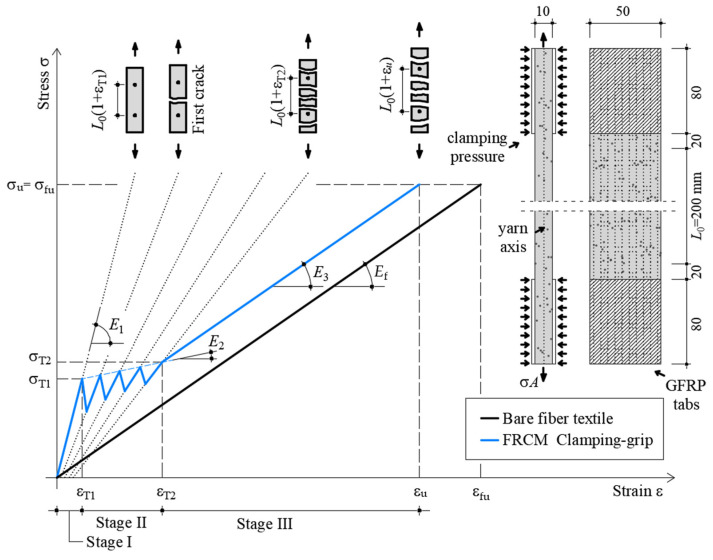
Idealized stress–strain response of quasi-static tensile test of FRCM coupons (dimensions in mm).

Specimens were tested adopting the clamping-grip tensile test configuration [19] (Figure 1), with the applied load aligned along the warp direction of the textile. The coupon ends were clamped by the testing machine wedges, which applied pressure to prevent slippage of the textile within the gripped length as required by [22]. In order to promote an even distribution of the clamping pressure and avoid mortar failure within the wedges, specimens were equipped with GFRP tabs of dimension 80 (length) × 50 (width), × 2 (thickness) mm bonded to the coupon ends (see Figure 1), which resulted in an overall specimen free length of 240 mm. An extensometer was applied to the central part of the specimen to measure the axial strain (see Figure 1). An *L*_0_ = 200 mm gauge length was adopted for the extensometer in an attempt to capture the majority of matrix cracks during the test. It should be noted that the clamping pressure induces stress concentrations that affect the matrix-fiber stress transfer mechanism along the gripping length [24]. However, this effect is not present along the specimen free length, where the deformation is measured. Indeed, the extensometer knife edges were placed 20 mm far from the end of the gripping portions to avoid interferences between the machine clamping pressure and the extensometer reading.

The specimens were subdivided into the following groups:

- Eight specimens were tested in quasi-static monotonic displacement-controlled loading conditions, at a rate of 0.2 mm/min, to evaluate the composite tensile properties;
- Five specimens were subjected to high-cycle fatigue tests, with applied load ranging between 35% and 3.5% of the quasi-static capacity of the 8 specimens tested before, with a frequency of 2 Hz. The test was initially conducted in quasi-static mode at a 0.2 mm/min displacement rate until reaching the maximum fatigue load. Then, the test was switched in force control, and the cyclic stage was started until attaining failure of the specimen. The initial quasi-static test was not interrupted at the mean fatigue load to avoid possible dynamic crack opening during the first cycle;
- Fifteen specimens were subjected to low-cycle fatigue tests, with applied load ranging between 75% and 5% of the corresponding quasi-static tensile capacity at a frequency of 1 Hz. Specimens were divided into 3 sets subjected to 5, 10, or 15 cycles, respectively. Initially, specimens were subjected to the quasi-static loading condition described before until the attainment of the mean fatigue load. Then, fatigue cycles were executed in load control mode. If failure was not attained at the end of the cycles, specimens were finally subjected to a quasi-static displacement-controlled test (with the same parameters of quasi-static tests described before) until failure.

Specimens were named according to the notation T_M(xx)_n, where T (= tensile test) indicates the test method, M indicates the test type (QS = quasi static, HF = high-cycle fatigue, LF = low-cycle fatigue), (xx) (if present) indicates the number of cycles in the low-cycle fatigue phase (xx = 5, 10, or 15), and n indicates the specimen number.

## 3. Results

Figure 1 reports the idealized stress σ–strain ε response of an FRCM coupon subjected to the clamping-grip tensile test [19], where σ = *P*/*A* is the axial stress, computed as the ratio between the load applied by the testing machine, *P*, and the fiber cross-sectional area, *A*.

The idealized stress–strain response shows a trilinear behavior [19]. During the first branch (Stage I in Figure 1), the specimen is not cracked, and its axial behavior is governed mainly by the matrix mechanical properties, which determine a slope of the stress–strain response, *E*_1_, consistent with the matrix elastic modulus. Stage I ends once the matrix tensile strength, σ_T1_ (Figure 1), is reached, corresponding to the strain ε_T1_. Beyond this point, the tensile stress is carried only by the fibers where the matrix is cracked, while it is shared between matrix and fibers in between consecutive cracks. During this stage (Stage II), new matrix cracks occur until matrix crack saturation is attained. The formation of new cracks during Stage II determines sudden drops in the load response that are associated with a decrease in the stress–strain curve slope (see dotted lines in Figure 1) and with a sudden increase in axial strain ε. It should be noted that dotted lines in Figure 1 do not represent possible unloading paths due to the non-linear specimen behavior. During this stage, the applied stress only slightly increases, which determines a limited slope of the idealized stress–strain curve *E*_2_. When no further matrix crack can occur [24], Stage II ends at an axial stress σ_T2_ and corresponding axial strain ε_T2_. During the following stage, named Stage III, the contribution of the matrix to the axial capacity of the specimen is quite small with respect to that of the textile, and the σ–ε response shows a slope *E*_3_ similar to that of the bare textile (*E*_f_). The specimen failure occurs once the fiber tensile strength, σ_fu_, is reached, or even before when an uneven distribution of stresses in the longitudinal fiber bundles occurs [25].

**Figure 2:**
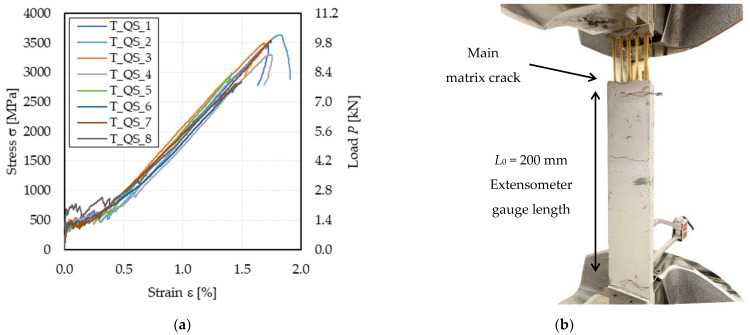
Quasi-static tests: (**a**) stress (load)–strain curves; (**b**) failure of specimen T_QS_1.

Table 2 reports the main parameters from the stress–strain responses of specimens subjected to the quasi-static test, shown in Figure 2a. In Table 2, *P*_u_ is the peak load observed, σ_u_, ε_u_, and *E*_3_ are the specimen tensile strength, ultimate strain, and stress–strain response slope during Stage III, respectively (see Figure 1). *E*_1_ was computed as the secant modulus between the initial point of the curve and point (σ_T1_; ε_T1_). The secant modulus *E*_2_ was assumed as the slope of the line between points (σ_T1_; ε_T1_) and (σ_T2_; ε_T2_). *E*_3_ was computed by linear regression of the point within 0.6σ_u_ and 0.9σ_u_ [26].

All tested specimens failed due to fiber rupture with cracked matrix, as shown in Figure 2b. The average FRCM cracking stress obtained, i.e., σ_T1_ = 440 MPa, which corresponds to an applied load *P* = 1.23 kN, is associated with a stress σ_m1_ = 2.46 MPa in the matrix, computed dividing the applied load by the matrix nominal cross-sectional area A_m_ = 500 mm^2^. σ_m1_ is 34% lower than the matrix tensile strength σ_mu_ provided in [24] (see Table 1). This difference can be attributed to the different test configurations adopted to obtain σ_m1_ (direct tensile test) and σ_mu_ (flexural and splitting tests [24]) and to the variability of the results commonly observed in tensile tests of quasi-brittle materials [27].

Since during Stage I, the contribution of the fiber textile to the applied load can be neglected, the slope of the axial stress σ_m_–axial strain ε curve during this stage, *E*_m1_, where σ_m_ = *P*/A_m_ is the matrix stress, can be compared with the matrix elastic modulus *E*_m_ [19]. The matrix elastic modulus *E*_m1_ computed in this way from Stage I resulted 64% higher than *E*_m_ (see Table 1). High values of *E*_m1_ were previously reported in the literature for various FRCM composites and can be attributed to uncertainties in the elastic modulus *E*_m_ declared by the manufacturer [28,29]. The stress–strain response slope in the cracked stage, also referred to as the cracked elastic modulus [26], *E*_3_, resulted on average 12% higher than that of bare textile strips (Table 1). This can be attributed to the fact that some cracks occurred outside the extensometer gauge length, which then measured an elongation not representative of the entire specimen [24]. In the cases where the main matrix cracks, i.e., the crack where fiber rupture was located, occurred outside the extensometer gauge length (see Figure 2b), the strain measured decreased after the peak load, which can be attributed to the elastic deformation recovery of the specimen portion where the extensometer was applied. The tensile strength of tested specimens resulted on average 10% lower than that of bare textile strips, which indicates that in most of the specimen the applied load was not evenly distributed among the textile bundles and among individual fiber filaments within each bundle.

**Figure 3:**
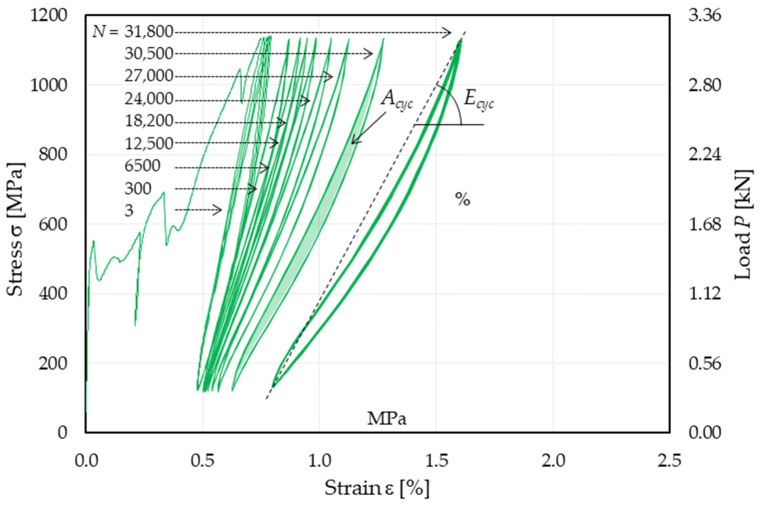
Stress (load)–strain response of specimen T_HF_2.

**Table 3:** Results of high-cycle fatigue test.

Specimen	*F_max_*	σ*_max_*	*F_min_*	σ*_min_*	*R*	N_F_
	[kN]	[MPa]	[kN]	[MPa]	[-]	[-]
T_HF_1	3.139	1121	0.406	145	0.13	25,143
T_HF_2	3.181	1136	0.334	119	0.10	31,842
T_HF_3	3.203	1144	0.319	114	0.10	17,036
T_HF_4	3.206	1145	0.318	114	0.10	32,898
T_HF_5	3.175	1134	0.330	118	0.10	40,807
Average	3.181	1136	0.341	122	–	29,545
CoV	0.85%		10.76%		–	30.24%

### 3.1. Behavior under High-Cycle Fatigue

The five specimens analyzed in this study showed a qualitatively similar stress–strain behavior under high-cycle fatigue loading. The key parameters of each test are reported in Table 3. All specimens failed due to fiber rupture within the extensometer gauge length before attaining the limit number of cycles (2 million) provided by the EAD for FRCM composites [22]. Although the same failure mode occurred for all specimens, the number of cycles at failure N_F_ was different (see Table 3), and a large dispersion was noted in the specimen fatigue lives (CoV = 30.24%, Table 3). This large scatter is consistent with that observed in quasi-brittle materials subjected to fatigue tests [27]. In Table 3, the values of maximum and minimum fatigue stress, namely σ*_max_* and σ*_min_*, respectively, and the corresponding stress ratio *R* = σ*_min_*/σ*_max_* are also reported. These values were slightly different from the nominal values (σ*_max_* = 1143 MPa and σ*_min_* = 114 MPa) due to difficulties of the machine load-control mode in matching the command with the specimen response, which varied during the test.

Figure 3 shows the stress (load)–strain response of specimen T_HF_2, which is representative of the behavior observed in specimens subjected to high-cycle fatigue. The response in Figure 3 can be divided into three parts:

- Response associated with the initial quasi-static monotonic test;
- Representative 10-cycle blocks selected at different numbers of cycles, namely N = 3, 300, 6500, 12,500, 18,200, 24,000, 27,500, 30,500, 31,800. The distance between the 10-cycle blocks varied throughout the cyclic process due to the residual inelastic strain accumulated;
- σ–ε response of the 10 cycles preceding failure of the specimen.

The initial quasi-static monotonic response of all T_HF specimens was consistent with that of specimens T_QS. Since the average fatigue load fell within Stage II of the idealized trilinear quasi-static response (see Figure 1 and Figure 2a), the quasi-static loading was interrupted at the attainment of the maximum fatigue stress σ*_max_* to avoid possible dynamic matrix cracks during the first cycle. During the fatigue test, a gradual reduction in the stress–strain curve slope was observed, alongside an increase in the cyclic area, which was associated with the progressive damaging and rupture of fiber filaments. Failure eventually occurred due to the reduction in fiber cross-sectional area, which made the composite no longer able to withstand the applied load.

The load cycles presented a peculiar shape. Starting from σ*_min_*, as the applied stress increases (left path of cycles in Figure 3), the derivative of the σ–ε curve (dσ/dε) decreases up to the mean stress value and then increases until attaining σ*_max_*. As the applied stress decreases from σ*_max_* (right path of cycles in Figure 3), dσ/dε decreases until attaining the mean stress value and then increases again until the cycle is concluded. This variation in cycle concavity, which was previously observed in high-cycle fatigue single-lap direct shear tests of FRP- and FRCM-concrete joints [30,31], could be attributed to the combined effect of loading rate and presence of friction/interlocking at the interface where debonding occurs (i.e., the FRP-concrete interface in FRP-concrete joints and the matrix-fiber interface in the FRCM studied in this paper). Indeed, the mechanical behavior of FRP and FRCM composites was observed to be affected by the loading rate [32,33], which varies during the cycle, and the presence of friction at the interface where debonding occurs affects the stress transfer mechanism both during the loading and unloading paths. Further studies are required to confirm these effects and fully understand the cyclic behavior of FRP and FRCM.

### 3.2. Behaviour under Low-Cycle Fatigue and Post-Cyclic Behaviour

Three sets of five specimens were subjected to 5, 10, and 15 low-cycle fatigue cycles, respectively. After the load cycles, if failure did not occur, a quasi-static test was performed, as described in Section 2, to determine the effect of low-cycle fatigue on the composite tensile properties. Table 4 reports the key parameters of the test performed, where N_PF_ is the number of load cycles performed and σ_u,PF_, ε_u,PF_, and *E*_3,PF_ are the (post-fatigue) tensile strength, ultimate strain, and cracked elastic modulus, respectively, evaluated on the post-fatigue quasi-static phase of the test as described in Section 3 for quasi-static tests.

**Figure 4:**
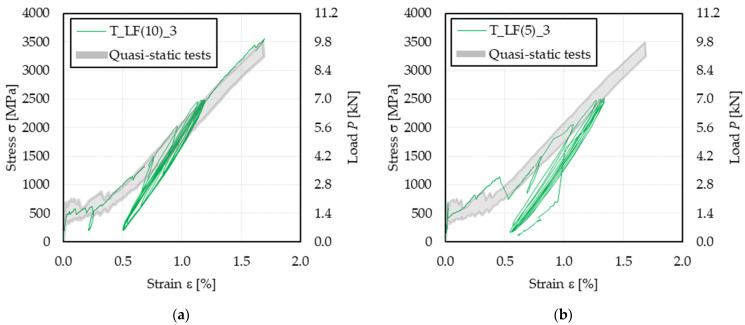
Stress (load)–strain curve of specimens subjected to low-cycle fatigue: (**a**) specimen T_LF(10)_3; (**b**) specimen T_LF(5)_3.

All specimens except T_LF(5)_3 did not fail during the low-cycle fatigue loading. A representative stress–strain response (specimen T_LF(10)_3) of specimens that did not fail due to fatigue loading is reported in Figure 4a, which includes the initial quasi-static phase, the low-cycle fatigue phase, and the post-fatigue quasi-static phase. At the end of the first quasi-static phase (i.e., when the mean fatigue stress was attained), specimens had already entered their fully-cracked stage (Stage III in Figure 1). Accordingly, their behavior during the low-cycle fatigue phase was mainly governed by the behavior of the fiber textile, while the matrix-fiber interaction did not play a fundamental role. It should be noted that one half-cycle (i.e., a cycle with half of the target amplitude) was imposed on the specimen before starting with the first complete cycle (i.e., cycle with the target amplitude), as clearly visible in Figure 4a,b. The half-cycle, which was disregarded in this study, was automatically applied by the testing machine to tune the load control parameters without risking applying a stress higher than σ*_max_*.

The stress–strain response of specimen T_LF(5)_3, which failed due to fatigue loading, is reported in Figure 4b. In this case, the premature failure could be attributed to a particularly uneven stress distribution among the textile bundles.

After the last cycle (except for specimen T_LF(5)_3), the load was maintained at the mean fatigue value, and the test control switched to the displacement control mode. The post-fatigue response was initially consistent with the last cycle. Then, beyond σ*_max_*, the stress–strain response resembled that of the quasi-static specimens T_QS, where the slope gradually decreased with increasing applied stress. Eventually, failure occurred due to textile rupture, which in all the cases took place outside the extensometer gauge length (see Figure 2b).

The load cycles of specimens subjected to low-cycle fatigue tests were characterized by a constant decreasing of dσ/dε during the loading (left path of cycles in Figure 4) and unloading (right path of cycles in Figure 4) paths. The difference between this shape and that of high-cycle fatigue tests (see Section 3.1) could be attributed to a less significant effect of loading rate and interface friction in specimens T_LF than in specimens T_HF. In low-cycle fatigue tests, the loading rate was lower than that of high-cycle fatigue tests, which entailed for a lower variation in loading rate during the cycle. Furthermore, due to the limited number of cycles, the contribution of friction to the applied load with respect to that of the bond stress transfer mechanism in specimens T_LF should be lower than that observed in specimens T_HF, where the high number of cycles may damage the matrix-fiber interface limiting the contribution of the bond stress transfer mechanism. Finally, specimen settling was observed during the first loading cycles, which may have affected their shape [31]. These observations were confirmed by the shape of initial cycles of high-cycle fatigue tests, which presented a less marked variation in concavity with respect to cycles after N = 500 (Figure 3).

Table 4 provides the percentage of tensile strength, ultimate strain, and cracked elastic modulus retained after the low-cycle fatigue, computed as the ratio between the average parameter measured after the low-cycle fatigue and the corresponding average parameter of specimens T_QS. According to the obtained results, a clear relationship between the number of load cycles and the parameters considered was not identified. The tensile strength σ_u,PF_ was almost unaffected by the fatigue loading for specimens subjected to either 5, 10, or 15 load cycles, which on average exhibited differences within −2% and 4%. However, the average values fall within the variability of observed tensile strengths of specimens T_QS (CoV = 9.56%, see Table 3). A similar observation can be made regarding the ultimate strain ε_u,PF_. Indeed, the three sets of specimens showed an average ultimate strain slightly higher than that of specimens T_QS (difference of 13%, 5%, and 9% for 5, 10, and 15 load cycles, respectively). Besides, all specimens subjected to low-cycle fatigue provided an average cracked elastic modulus *E*_3,PF_ higher than that of corresponding quasi-static specimens, although a large variability was observed. Namely, specimens subjected to 5, 10, and 15 cycles provided an average *E*_3,PF_ 14%, 24%, and 15%, respectively, higher than that of T_QS specimens. This result can be explained by the position of the main matrix crack, which was always located outside the extensometer gauge length, may be as a result of the stress concentration caused by the clamping pressure.

## 4. Discussion

### 4.1. High-Cycle Fatigue Tests

**Figure 5:**
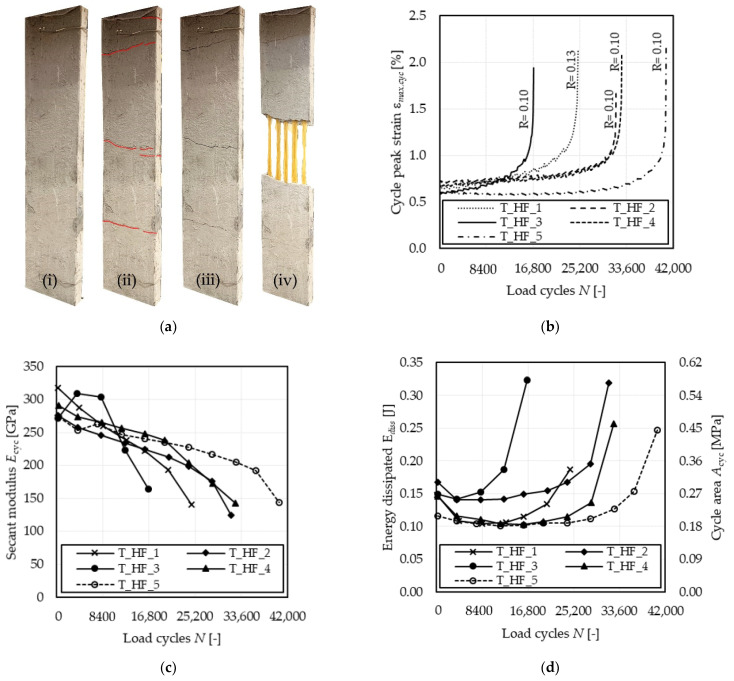
Effect of high-cycle fatigue on the tensile response of tested specimens. (**a**) Crack pattern during the test: (**i**) beginning of the test, (**ii**) end of the quasi-static phase, (**iii**) cyclic phase, (**iv**) failure. (**b**) Variation in cycle peak strain with the number of cycles. (**c**) Variation in the secant modulus with the number of cycles. (**d**) Variation in energy dissipated (cycle area) with the number of cycles.

In Figure 5, the influence of high-cycle fatigue on the tensile performance of the tested specimens is investigated. Three parameters of the stress–strain response were analyzed; namely, the axial strain corresponding to the maximum applied stress of each cycle ε*_max,cyc_*, the area enclosed within the cyclic response, *A_cyc_*, and the cycle secant modulus (see Figure 3), *E_cyc_*. *E_cyc_* was computed as the slope of the straight line connecting two subsequent peak and valley points of the loading cycle [34].

#### 4.1.1. Strain and Crack Growth

Figure 5a,b show the development of the crack pattern during the test and the cycle peak strain (ε*_max,cyc_*) growth over the cyclic phase, respectively. The quasi-static phase ended once the nominal load value σ*_max_* = 1143 MPa was attained, which was associated with Stage III of the quasi-static response. Accordingly, at the end of the quasi-static phase of the test, all the cracks in the matrix were already formed (Figure 5a(ii)). Then, during the cyclic phase of the test, no new cracks occurred (Figure 5a(iii)) while the cracks opened in the preceding phase started widening and propagating with an increasing number of cycles. A stable increase in the cycle peak strain, which was associated with the widening of existing cracks, was observed in all tests (Figure 5b). When the cycle peak strain growth became unstable, one of the cracks (referred to as main matrix crack, usually located within the extensometer gauge length) started widening at a higher rate with respect to the others due to local damaging and rupture of fiber filaments. The specimen failure occurred at the main matrix crack once the residual (undamaged) fibers were no longer able to withstand the applied load. Failure of the specimens was characterized by telescopic failure of fiber bundles, which determined fiber filament rupture at different locations instead of a localized sharp failure (Figure 5a(iv)).

#### 4.1.2. Secant Modulus

All specimens subjected to high-cycle fatigue showed a variation in their axial stiffness during the test. In this study, the cycle secant modulus *E_cyc_* (see Figure 3) was considered to analyze the composite axial stiffness variation.

Figure 5c shows the variation in *E_cyc_* for the specimens tested, computed as the average of the secant modulus of 10 consecutive cycles extracted approximatively each 4000 load cycles (each marker in Figure 5c represents an *E_cyc_* computed in this way). All the specimens exhibited an initial *E_cyc_* from 39% to 62% higher than the bare textile elastic modulus (see Table 1), due also to the presence of cracks outside the extensometer gauge length (see Figure 5a). The majority of the specimens showed an almost linear reduction in the cycle secant modulus with the number of cycles, which can be associated with progressive rupture of fiber filaments in the textile bundles. Only for specimen T_HF_3, an initial *E_cyc_* increase was recorded within the first 4000 cycles, which can be attributed to the initial widening of a crack outside the extensometer gauge length. When failure occurred, *E_cyc_* was between 130 and 160 GPa for the specimens tested, corresponding to 66% and 82% of *E*_f_, respectively, which indicates a diffused damage of fiber and matrix if compared with *E*_3_ of quasi-static specimens (see Table 2).

#### 4.1.3. Energy Dissipated

A parameter of great interest in the study of the fatigue behavior is the energy dissipated during the fatigue cycles, E*_diss_*, which is proportional to the area enclosed within each load cycle of the stress–strain response (*A_cyc_* in Figure 3), according to the equation:

(1) $$E_{diss}=A_{cyc}AL_{0}$$

where *A* is the total fiber cross-sectional area and *L*_0_ is the extensometer gauge length (see Figure 2b). Figure 5d shows the variation in E*_diss_* for the specimens tested, computed as the average of the dissipated energy of 10 consecutive cycles extracted approximatively each 4000 load cycles. The five specimens analyzed showed a qualitatively similar behavior, with an initial decreasing trend that tended to stabilize to an approximately constant value, which was then maintained during the steady strain growth phase (see Figure 5b). Once the strain growth became unstable, ε*_max,cyc_* rapidly increased, and the cycle area became larger. During the test, both the cycle peak (ε*_max,cyc_*) and valley (ε*_min,cyc_*) strain increased. ε*_max,cyc_* increased with a higher rate with respect to ε*_min,cyc_*, which explains the decrease in stiffness observed. In addition, the shape of cycles changed during the test. The first cycles were characterized by a larger difference between deformations at the mean applied stress of the ascending and descending branches with respect to the steady phase cycles (see Figure 3), which determined high dissipated energy at the beginning of the cycle phase (Figure 5d). This phenomenon is attributed to the specimen stabilization during the first cycles.

### 4.2. Low-Cycle Fatigue Tests

**Figure 6:**
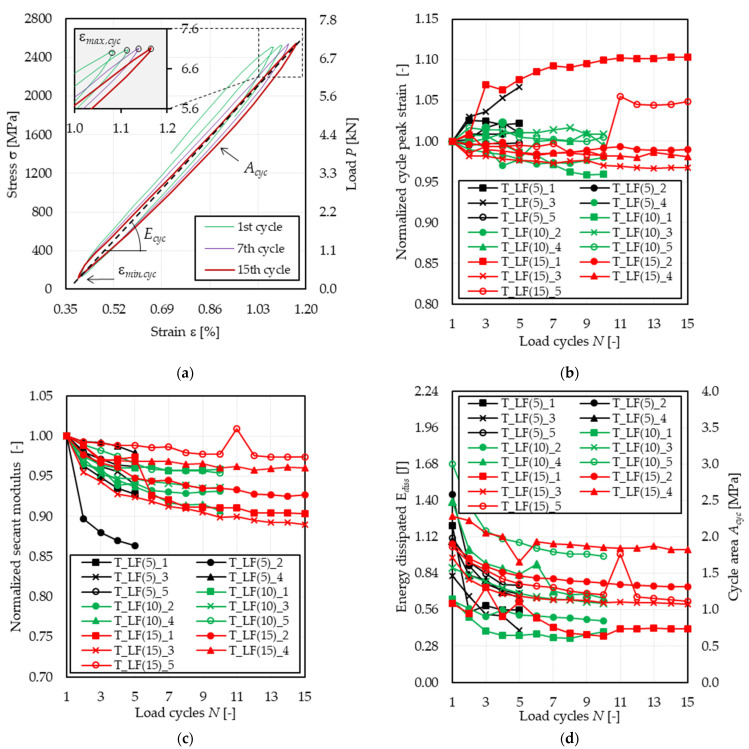
Effect of low-cycle fatigue on the tensile response of tested specimens. (**a**) Stress (load)–strain response of specimen T_LF(15)_3 during the cyclic phase. (**b**) Variation in the normalized cycle peak strain with the number of cycles. (**c**) Variation in the normalized secant modulus with the number of cycles. (**d**) Variation in the energy dissipated (cycle area) with the number of cycles.

The effect of low-cycle fatigue on the tensile response of the PBO FRCM composite analyzed in this study is investigated in Figure 6 with respect to the parameters presented in Figure 6a, namely the cycle peak strain ε*_max,cyc_*, the area enclosed within the load cycles *A_cyc_*, and the cycle secant modulus *E_cyc_*. In particular, Figure 6a shows the stress (load)–strain response of specimen T_LF(15)_3 corresponding to the first, seventh, and fifteenth (last) load cycle. In Figure 6a, the cycle area is computed as the surface enclosed by the stress–strain curve within two consecutive valley points, whereas the cycle secant modulus is the slope of the straight line joining two subsequent peak and valley points of the stress (load) cycle.

Due to the high mean stress and amplitude considered in low-cycle fatigue tests, the first cycle strongly affected the specimen behavior, which in turn determined a relevant scatter of ε*_max,cyc_* and *E_cyc_* obtained by each specimen. Therefore, to compare the results of these specimens, in Figure 6b,c, the parameters studied were normalized with respect to the values they assumed in the first cycle. This was not necessary in the case of high-cycle fatigue tests since the mean stress and amplitude were lower than those of low-cycle fatigue tests and also because σ*_max_* was attained in the quasi-static phase preceding the beginning of the fatigue phase.

#### 4.2.1. Strain and Crack Growth

It should be noted that the mean fatigue load is associated with the cracked stage of the specimen (Stage II in Figure 1). Accordingly, the crack pattern was fully developed at the beginning of the cyclic phase. Figure 6b shows the variation in the specimen axial strain corresponding to the maximum applied stress of each load cycle ε*_max,cyc_* (see Figure 6a), normalized with respect to the ε*_max,cyc_* value measured in the first load cycle. For the majority of the tested specimens, ε*_max,cyc_* remained approximately constant throughout the whole cyclic phase, with a variation ranging between 95% and 105% of ε*_max,cyc_* of the first cycle. Specimen T_LF(5)_3 exhibited the highest rate of increase in ε*_max,cyc_*, which could be attributed to the occurrence of fiber damage at cracks located within the extensometer gauge length. It should be noted that the strain associated with the cycle valley, ε*_min,cyc_* (see Figure 6a), did not vary during the cyclic phase (see Figure 6a). This indicates that the specimens were able to fully recover the axial strain induced during the load cycle, which in turn confirms the limited damage induced by low-cycle fatigue. The only exception was specimen T_LF(5)_3, which in fact, failed during the cyclic phase. In specimen T_LF(15)_5, the sudden opening of a matrix crack within the extensometer gauge length during the tenth cycle determined a sudden increase in the cycle peak strain, as shown in Figure 6b. This also affected the secant modulus and energy dissipated, which showed a sudden increase for the point following the tenth cycle.

Differently from high-cycle fatigue, the low-cycle fatigue peak strain growth generally tended to stabilize or reduce, which can be justified by the fact that in high-cycle fatigue, the main crack occurred within the extensometer gauge length, whereas for specimens in series T_LF, it occurred outside the extensometer gauge length. This was also confirmed in the case of specimen T_LF(5)_3, which, although failed during the cyclic phase as all the T_HF specimens, showed a main crack outside the extensometer gauge length. This suggests that different fatigue mean stresses and amplitudes have an influence on the failure mode. In high-cycle fatigue tests, failure is caused by progressive damage of fiber filaments, which is induced by continuous friction with the embedding matrix. In low-cycle fatigue tests, where a high value of the maximum applied load is considered, failure is similar to that observed in quasi-static tests where more fiber filaments fail at the same time.

#### 4.2.2. Secant Modulus

The secant modulus *E_cyc_* can be considered to study the axial stiffness variation in the specimens during the test. In Figure 6c, *E_cyc_* of each cycle was normalized with respect to *E_cyc_* of the first cycle to provide an indication of the stiffness degradation during the fatigue phase. Figure 6c shows that, although *E_cyc_* decreased for all specimens, the maximum reduction with respect to *E_cyc_* of the first cycle was lower than 13% (specimen T_LF(5)_2). This observation is consistent with the observations of limited and no variation in cycle peak and valley strain, ε*_max,cyc_* and ε*_min,cyc_*, respectively, which entailed a limited variation in *E_cyc_*. It should be noted, however, that in the case of low-cycle fatigue tests, the main crack was usually located outside the extensometer gauge length. Therefore, the measure of *E_cyc_* may not be representative of the actual behavior of the specimens.

#### 4.2.3. Energy Dissipated

The energy dissipated during low-cycle fatigue tests was much higher than that dissipated during high-cycle fatigue tests, due mainly to the different cycle amplitude and mean stress values. All specimens showed a similar trend of dissipated energy during the cycles (Figure 6d). During the first cycles, the specimens stabilized, and the area of each cycle tended to reduce to an approximately constant value, as observed in the case of high-cycle fatigue tests (Figure 5d). For specimen T_LF(5)_3, the dissipated energy did not tend to a constant value but kept decreasing during the cycles until failure of the specimen. This suggests that great damage occurred during the first cycles, which hindered the possibility of a stable fatigue phase leading instead to premature failure.

While in high-cycle fatigue tests, the energy dissipated E*_diss_* first decreased and then increased with increasing the number of cycles (see Figure 5d), in low-cycle fatigue tests, E*_diss_* monotonically decreased (except for some points) during the fatigue phase. However, since for both high-cycle and low-cycle fatigue tests, E*_diss_* decreased during the first cycles, the results obtained did not allow for identifying a clear effect of the load level on the variation in the dissipated energy during the fatigue phase.

## 5. Conclusions

In this paper, the tensile behavior of PBO FRCM composite coupons subjected to fatigue (cyclic) loading was investigated. First, eight specimens were subjected to displacement-controlled quasi-static tests. Then, five specimens were subjected to a high-cycle fatigue test with load ranging between 3.5% and 35% of the average tensile capacity obtained by quasi-static tests. Finally, 15 specimens were subjected to a low-cycle fatigue test with load ranging between 5% and 75% of the corresponding quasi-static tensile capacity. All tests were conducted using the clamping-grip method. The results obtained allowed for drawing the following main conclusions:

- Specimens subjected to quasi-static tests showed limited result scatter and provided load responses consistent with the idealized trilinear load response provided for FRCM composites. The failure occurred due to textile rupture, usually outside the extensometer gauge length;
- The fatigue life of specimens subjected to high-cycle fatigue was always lower than 33,000 cycles. Failure occurred due to progressive fiber filaments rupture at the main matrix crack located within the extensometer gauge length. This allowed for studying the evolution of the axial strain, axial stiffness, and energy dissipated during the cyclic phase. The axial strain and energy dissipated showed a stable phase during the test, whereas they rapidly increased during the final unstable fatigue phase. The axial stiffness decreased following an approximately linear trend during the entire fatigue test;
- Specimens subjected to low-cycle fatigue showed failure due to textile rupture at a main matrix crack outside the extensometer gauge length. This affected the study of axial strain, axial stiffness, and energy dissipated during the fatigue phase. After an initial increase, the axial strain stabilized and kept an approximately constant value during the test. The axial stiffness decreased during the first cycles and then tended to stabilize to a constant value. Similarly, the energy dissipated decreased during the first cycles and generally approached an approximately constant value during the following cycles. When subjected to a post-fatigue quasi-static tensile test, the specimens showed a limited reduction in the tensile properties with respect to quasi-static specimens not previously subjected to fatigue loading.

The results presented in this paper provide important indications on the tensile behavior of PBO FRCM composite coupons subjected to high- and low-cycle fatigue tests. However, further studies are needed to investigate the properties of the composite when externally bonded to existing concrete and masonry members subjected to fatigue loading.

## Figures and Tables

**Table 1:** Mechanical properties of fiber textile and matrix.

Property	PBO Textile (Warp)	Matrix
Tensile strength—σ_fu_, σ_mu_	3620 MPa	3.75 MPa ^¤^
Elastic modulus—*E*_f_, *E*_m_	196 GPa	7.0 GPa ^¤^
Ultimate strain—ε_fu_	1.85%	–
Compressive strength	–	51.60 MPa
Flexural strength	–	8.10 MPa

^¤^ Taken from [24].

**Table 2:** Results of quasi-static tensile test on FRCM coupons.

Specimen	σ_T1_ [MPa]	ε_T1_ [%]	*E*_1_ [GPa]	σ_T2_ [MPa]	ε_T2_ [%]	*E*_2_ [GPa]	*P*_u_ [kN]	σ_u_ [MPa]	ε_u_ [%]	*E*_3_ [GPa]
T_QS_1	376	0.031	980	820	0.415	116	9.738	3478	1.726	218
T_QS_2	381	0.024	1317	574	0.353	59	10.189	3639	1.819	218
T_QS_3	407	0.017	1978	752	0.392	92	9.814	3505	1.688	213
T_QS_4	450	0.019	1831	638	0.357	56	9.254	3305	1.728	227
T_QS_5	443	0.041	995	671	0.389	66	8.362	2986	1.417	232
T_QS_6	459	0.011	3988	530	0.197	47	7.868	2810	1.452	219
T_QS_7	337	0.020	1506	557	0.281	84	9.910	3539	1.744	216
T_QS_8	670	0.017	3864	860	0.378	52	7.969	2846	1.499	214
Average	440	0.023	2057	675	0.345	71	9.138	3264	1.634	220
CoV	21.66%	39.70%	54.86%	17.19%	19.56%	31.26%	9.56%	9.56%	8.80%	2.83%

**Table 4:** Results of low-cycle fatigue tests.

Specimen	*F_max_*	σ*_max_*	*F_min_*	σ*_min_*	*R*	N_PF_	σ_u,PF_	ε_u,PF_	*E*_3,PF_
	[kN]	[MPa]	[kN]	[MPa]	[-]	[-]	[MPa]	[%]	[GPa]
T_LF(5)_1	7.097	2535	0.384	137	0.05	5	3043	1.42	350
T_LF(5)_2	7.121	2543	0.328	117	0.05	5	3633	1.66	238
T_LF(5)_3 ^†^	7.022	2508	0.316	166	0.05	5	–	–	–
T_LF(5)_4	7.084	2530	0.369	132	0.05	5	3605	1.91	232
T_LF(5)_5	7.084	2530	0.389	139	0.05	5	3271	2.37	179
Average	–	–	–	–	–	–	3388	1.84	250
CoV	–	–	–	–	–	–	8.35%	22.00%	28.77%
Retained	–	–	–	–	–	–	104%	113%	114%
T_LF(10)_1	6.437	2299	1.131	404	0.18	10	2898	1.57	282
T_LF(10)_2	6.642	2372	0.893	319	0.13	10	2551	0.96	424
T_LF(10)_3	6.969	2489	0.541	193	0.08	10	3552	1.70	259
T_LF(10)_4	7.082	2529	0.405	145	0.06	10	3397	2.04	227
T_LF(10)_5	7.122	2544	0.345	123	0.05	10	3626	2.35	175
Average	–	–	–	–	–	–	3205	1.72	274
CoV	–	–	–	–	–	–	14.44%	30.41%	34.11%
Retained	–	–	–	–	–	–	98%	105%	124%
T_LF(15)_1	6.715	2398	0.789	282	0.12	15	3582	1.76	256
T_LF(15)_2	7.091	2532	0.401	143	0.06	15	3217	1.57	298
T_LF(15)_3	7.127	2545	0.309	110	0.04	15	3357	1.56	283
T_LF(15)_4	7.132	2547	0.295	105	0.04	15	3588	2.14	210
T_LF(15)_5	7.114	2541	0.343	123	0.05	15	3254	1.89	215
Average	–	–	–	–	–	–	3400	1.78	252
CoV	–	–	–	–	–	–	5.21%	13.56%	15.67%
Retained	–	–	–	–	–	–	104%	109%	115%

^†^ Specimen failed during the low-cycle fatigue phase.

## Data Availability

The data presented in this study are available on request from the corresponding author. The data are not publicly available because the research is still ongoing.
